# The Microstructure of Nanocrystalline TiB_2_ Films Prepared by Chemical Vapor Deposition

**DOI:** 10.3390/ma10121425

**Published:** 2017-12-13

**Authors:** Xiaoxiao Huang, Shuchen Sun, Ganfeng Tu, Shuaidan Lu, Kuanhe Li, Xiaoping Zhu

**Affiliations:** School of Metallurgy, Northeastern University, No. 3-11, Wenhua Road, Heping District, Shenyang 110819, China; huangxiaoxiao@outlook.com (X.H.); tugf@smm.neu.edu.cn (G.T.); imiage@163.com (S.L.); kevinlee579@outlook.com (K.L.); xiaopingzhu007@outlook.com (X.Z.)

**Keywords:** TiB_2_, nanocrystalline films, microstructure, chemical vapor deposition

## Abstract

Nanocrystalline titanium diboride (TiB_2_) ceramics films were prepared on a high purity graphite substrate via chemical vapor deposition (CVD). The substrate was synthesized by a gas mixture of TiCl_4_, BCl_3_, and H_2_ under 1000 °C and 10 Pa. Properties and microstructures of TiB_2_ films were also examined. The as-deposited TiB_2_ films had a nano-sized grain structure and the grain size was around 60 nm, which was determined by X-ray diffraction, field emission scanning electron microscopy, and transmission electron microscopy. Further research found that a gas flow ratio of TiCl_4_/BCl_3_ had an influence on the film properties and microstructures. The analyzed results illustrated that the grain size of the TiB_2_ film obtained with a TiCl_4_/BCl_3_ gas flow ratio of 1, was larger than the grain size of the as-prepared TiB_2_ film prepared with a stoichiometric TiCl_4_/BCl_3_ gas flow ratio of 0.5. In addition, the films deposited faster at excessive TiCl_4_. However, under the condition of different TiCl_4_/BCl_3_ gas flow ratios, all of the as-prepared TiB_2_ films have a preferential orientation growth in the (100) direction.

## 1. Introduction

The titanium diboride (TiB_2_) is an interesting and very useful ceramic material. It displays many attractive properties such as high melting temperature, high hardness, high elastic modulus, erosion resistance, excellent chemical stability, and good thermal as well as electrical conductivity [1,2,3,4,5,6]. Thus, TiB_2_ is widely applied as the evaporator boat in high vacuum metal films, the protection of weapons and armored vehicle [7,8,9]. In addition, the neutron absorption of boron coupled with the above-high-temperature properties makes TiB_2_ the best choice of control rod material for high-temperature nuclear reactors [10,11,12].

The outstanding properties of TiB_2_ depend on its microstructure. TiB_2_ crystallizes with a hexagonal structure with a P6/mmm space group [13,14]. Titanium atoms are located at the vertex of the hexagonal prism and the center of the bottom, boron atoms fill the trigonal prisms that are formed by the titanium atoms, and the boron atom and the titanium atom alternately form a 2D honeycomb network structure. B–B is covalently bonded, and B–Ti is bounded by ion bonds [15,16]. The lattice parameters of TiB_2_ are as follows: a = b = 3.029, c = 3.228, α = β = π/2, and γ = π/3 [17,18].

Over the past few decades, many studies have been carried out on the deposition of TiB_2_ films by chemical vapor deposition (CVD) methods [19,20,21], plasma assisted chemical vapor deposition (PACVD) methods [6,11], and plasma enhanced chemical vapor deposition (PECVD) methods [22]. Takehiko Takahashi and Hideo Kamiya [21] investigated the influence of deposition temperature on the deposition phase using CVD methods, the results show that TiB_2_ could be deposited above 800 °C. A. J. Caputo et al. [19] have researched the effect of deposition temperature on film hardness, the surface morphology of the films, and deposition rate via CVD methods. The PACVD and PECVD technique have a low deposition temperature range of 250~650 °C. In this paper, nanocrystalline TiB_2_ films on a high pure graphite substrate were deposited at a higher temperature (1000 °C) by a CVD system. The purpose of our work was to investigate the deposition results and to prepare for further study in which TiB_2_ films are deposited on a nickel-based superalloy substrate. Because of the properties of nickel-based superalloys, deposition temperatures should not exceed 1000 °C. Thus, the deposition temperature was also limited to 1000 °C in this experiment. The structure of the TiB_2_ films was examined by X-ray diffraction (XRD), field emission scanning electron microscopy (FESEM), transmission electron microscopy (TEM), and energy dispersive spectroscopy (EDS). Furthermore, the TiB_2_ films were synthesized using different TiCl_4_/BCl_3_ gas flow ratios. The influence of the TiCl_4_/BCl_3_ gas flow ratio on the grain size and the deposition rate is discussed.

## 2. Experimental Methods

TiB_2_ films were deposited on a high pure graphite substrate via CVD using a gas mixture of TiCl_4_, BCl_3_, and H_2_. The overall reaction of the vapor deposition of TiB_2_ is as follows [21]:

TiCl_4_ (g) + 2BCl_3_ (g) + 5H_2_ (g) = TiB_2_ (s) + 10HCl (g). (1)

**Figure 1:**
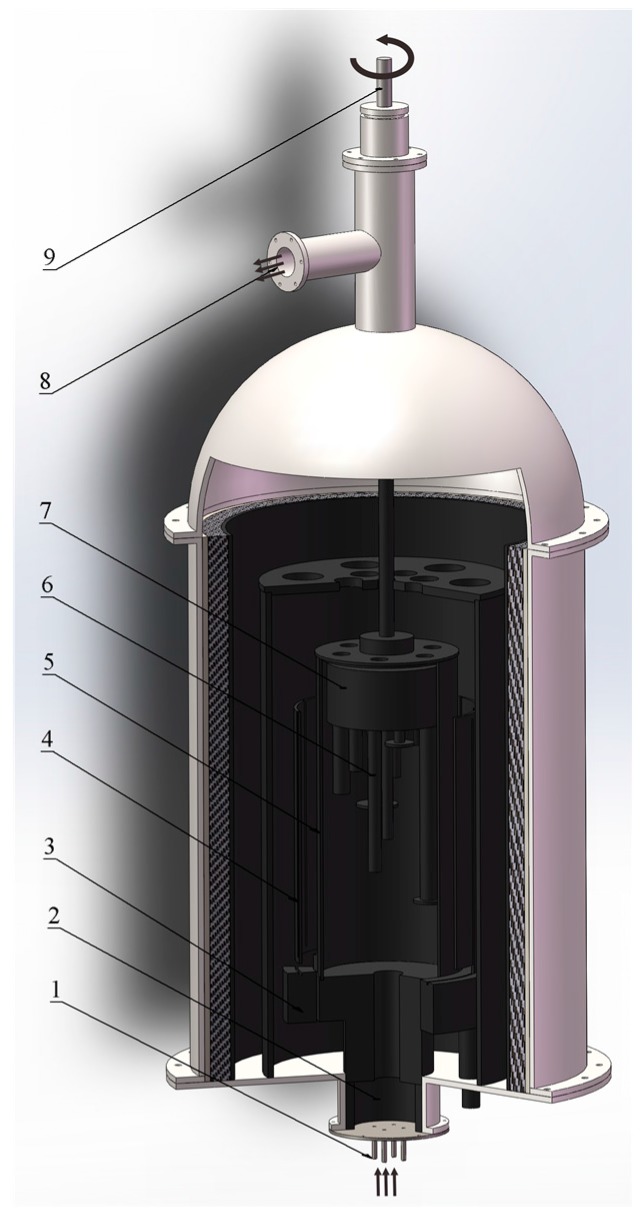
3D model diagram of the CVD (chemical vapor deposition) reactor. (**1**) Air inlet; (**2**) gas mixing chamber; (**3**) electrode; (**4**) heating resistor; (**5**) deposition chamber; (**6**) high purity graphite substrate; (**7**) sample stage; (**8**) air outlet; (**9**) rotating shaft.

The 3D model diagram of the CVD reactor is illustrated in Figure 1. BCl_3_, TiCl_4_, and H_2_ enter the deposition chamber through the air inlet at the bottom of the CVD reactor, however, H_2_, BCl_3_, and TiCl_4_ were mixed in the gas mixing chamber before importing the deposition chamber. The graphite substrates were fixed on the sample stage. When the reaction begins, the rotating shaft drives the sample table to drive the graphite substrate to rotate. The rotating graphite substrate ensures the uniform films. A vacuum pump is connected to the air outlet for the aim of keeping the reactor in a vacuum during this entire sequence. The vacuum level was maintained at about 10 Pa during the reaction. During experimentation, TiCl_4_ and BCl_3_ were carried to the reactor by a heated line. The temperature of the TiCl_4_ flow was 135 °C, and the BCl_3_ flow was 12 °C. The heated TiCl_4_ flow and BCl_3_ flow were controlled by a mass flowmeter. Table 1 summarizes the field of deposition parameters.

In this study, the deposition was carried out at 1000 °C. The deposition pressure was 10 Pa, and the deposition time was 3 h. In the experiment, the supply of hydrogen was excessive, and the BCl_3_ flow rate was kept at 0.085 m^3^/h, then two types of specimens were synthesized at two TiCl_4_ flow rate levels: 0.055 m^3^/h and 0.11 m^3^/h. When the TiCl_4_ flow rate was 0.055 m^3^/h, the gas flow ratio of TiCl_4_/BCl_3_ (κ) was 0.5. At this point, κ was the stoichiometric TiCl_4_/BCl_3_ gas ratio. When the TiCl_4_ flow rate was 0.11 m^3^/h, κ was 1. TiCl_4_ was excessive in this situation. The microstructure and properties of these two types of films were investigated. In this way, the influence of the TiCl_4_/BCl_3_ gas flow ratio on the grain size and the deposition rate was studied.

The crystal phase of the films was determined by XRD (X’Pert Pro, PANalytical B.V., Almelo, The Netherlands). Special emphasis was put on the comparative analysis of micro structural characterization of these two types of films. In addition, SEM (Ultra Plus, ZEISS, Heidenheim, Germany), TEM (Tecnai G^2^ 20, FEI, Hillsboro, OR, USA), and EDS (X-Max 50, OXFORD, Oxford, UK) were employed.

## 3. Results and Discussion

### 3.1. Structure, Morphology, and the Deposition Rate of Films

**Figure 2:**
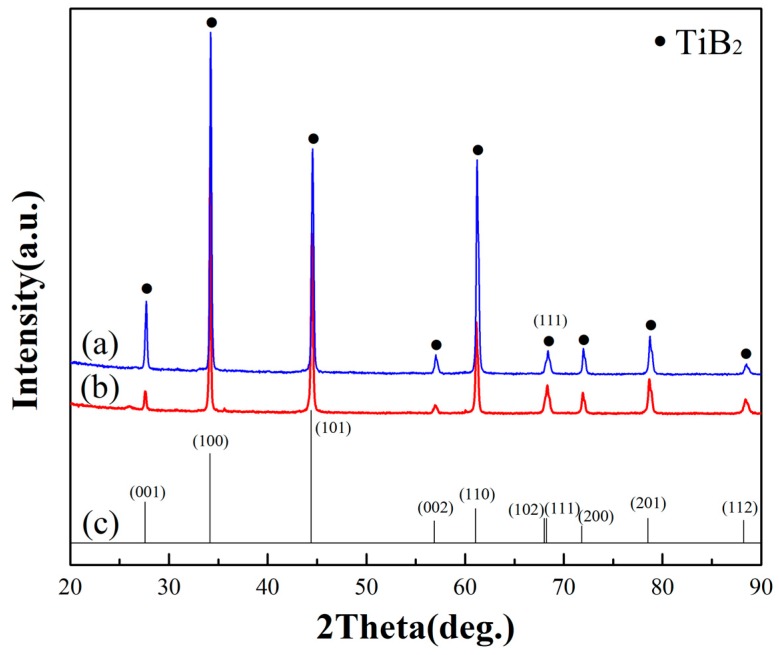
XRD (X-ray diffraction) patterns of TiB_2_ deposits. (**a**) At a gas flow ratio of TiCl_4_/BCl_3_ = 0.5; (**b**) at a gas flow ratio of TiCl_4_/BCl_3_ = 1; (**c**) TiB_2_ standard data from JCPDS (Joint Committee on Powder Diffraction Standards) card No. 85-2083.

It was found that the oriented growth was related to the ratio of TiCl_4_/BCl_3_ from the orientation characteristics of the deposited layers investigated by X-ray diffraction shown in Figure 2 for a laser power 3 kW and laser scanning speed 2.5 mm/s. Figure 2a,b show the diffraction patterns of the deposits, which were prepared at κ = 0.5 and 1, respectively. In addition, the TiB_2_ standard data from the Joint Committee on Powder Diffraction Standards (JCPDS) is shown in Figure 2c. X-ray diffraction (Figure 2a,b) indicated the presence of only TiB_2_, with no unidentified peaks. Figure 2 also shows that the diffraction pattern of deposits corresponds well to the JCPDS standard for TiB_2_. Furthermore, we found that the pattern intensity of the TiB_2_ films, which were deposited at different gas ratios of TiCl_4_/BCl_3_ (Figure 2a,b), follows a similar trend. The intensity of the (100) peaks in Figure 2a,b are the highest of all peaks, respectively. However, there is an enhanced intensity at the (100) peaks compared with the standard pattern intensity shown in the JCPDS card NO. 85-2083 (Figure 2c). Furthermore, according the XRD analysis, the relative intensity value of R_(100)/(101)_ is1.32 ± 0.01 (Figure 2a) and 1.28 ± 0.01 (Figure 2b). Similarly, the relative intensity of the standard pattern yields a R_(100)/(101)_ value of 0.86 ± 0.01 (Figure 2c). Obviously, the R_(100)/(101)_ values of the as-prepared TiB_2_ films are greater than the standard values. Hence, all the as-prepared TiB_2_ films have a preferred orientation along the (100) planes. These results are consistent with the work done by S.H. Lee [11], who reported that the film structure has a (100) preferred orientation when the film was deposited at a low RF power (200 W). The difference is that they prepared the TiB_2_ film at a low temperature (250~400 °C) using a PACVD system.

**Figure 3:**
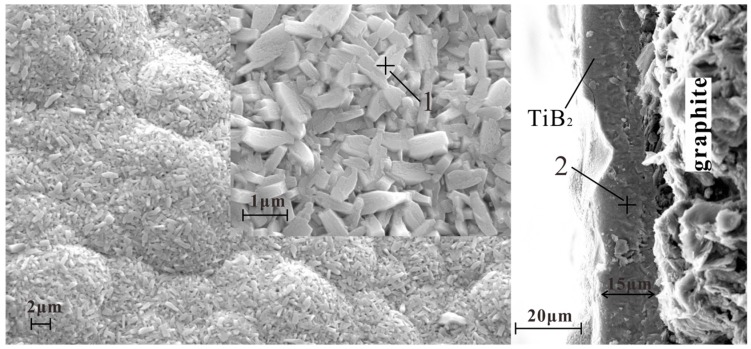

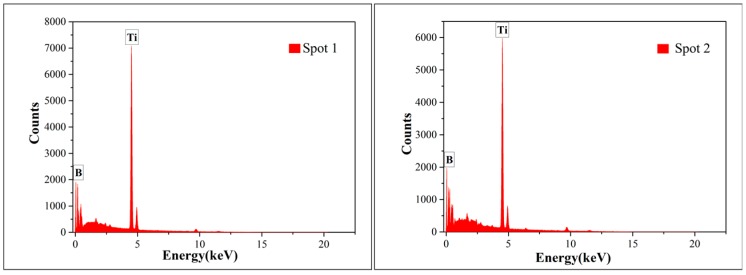
SEM (scanning electron microscopy) images and EDS (energy dispersive spectroscopy) results of the surface and cross section of the TiB_2_ film formed under the condition of a TiCl_4_/BCl_3_ gas flow ratio of 0.5.

**Figure 4:**
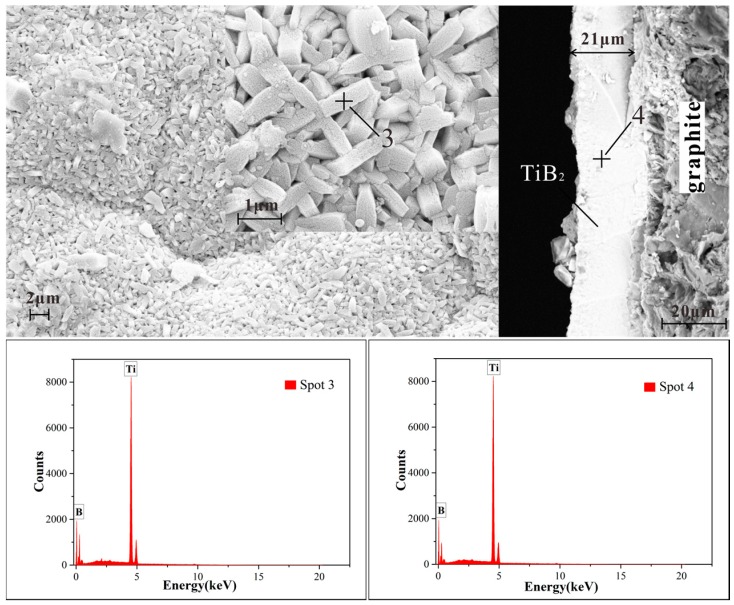
SEM images and EDS results of the surface and cross section of the TiB_2_ film formed under the condition of a TiCl_4_/BCl_3_ gas flow ratio of 1.

The surfaces and cross sections of the TiB_2_ films obtained by κ 0.5 and 1 were observed by FESEM (Figure 3 and Figure 4, respectively) with high voltages of 15 kV and magnifications of 5000× and 20,000×. EDS results are also shown. An unsmooth surface can be observed on the films. The cross sections show a very compact and dense structure without any gaps. Figure 4 indicates the FESEM images and EDS results of the surface and cross section of TiB_2_ films formed under the conditions of κ = 1. It can be discussed on different micrographs of the surfaces and cross sections by Figure 3 and Figure 4. It was found that similar morphologies of the TiB_2_ films were obtained by different gas flow ratios of TiCl_4_/BCl_3_. All TiB_2_ films were found to have a typically columnar particle structure, and the columnar particles have a random orientation. This indicates that there was no distinct effect on morphologies by the TiCl_4_/BCl_3_ gas flow ratio in this experimentation. However, different film thicknesses were displayed in the cross sections (Figure 3 and Figure 4). As shown in Figure 3, the thickness of the film deposited at κ = 0.5 for 3 h reached 15 microns. The thickness was 21 microns when κ was equal to 1 in Figure 4. Obviously, a thicker TiB_2_ film was obtained by a greater TiCl_4_/BCl_3_ gas flow ratio that goes beyond the stoichiometric TiCl_4_/BCl_3_ gas flow ratio. In other words, the film deposited faster when TiCl_4_ was excessive.

Table 2 shows the compositional analysis results of Spot 1, Spot 2, Spot 3, and Spot 4, analyzed via EDS. The Ti/B ratio of the deposit approached the stoichiometric value of 0.5 when the gas flow ratio of TiCl_4_/BCl_3_ was increased. According to the CVD phase diagram of the Ti–B–H–Cl system which was calculated by Randich and Gerlach [23], the formability of TiB_2_ relies on the distance between the process tie line and the phase boundary of the TiB_2_+ gas and the gas phases. A boron rich TiB_x_ film was deposited when the BCl_3_ concentration was high, because the process tie line went through the region of the B + TiB_2_ + gas phase. Therefore, at a low TiCl_4_/BCl_3_ gas ratio, boron rich TiB_x_ films were formed instead of TiB_2_.

### 3.2. Grain Size of the Films

Many scholars [6,11,19] have researched the influence of deposition temperature on grain size, and they all agreed that the grain size increases with increasing temperature. Indeed, this conclusion is in good agreement with the kinetic theory of CVD [24]. Comparatively few research groups have studied the effect of TiCl_4_/BCl_3_ gas flow ratio on grain size.

**Figure 5:**
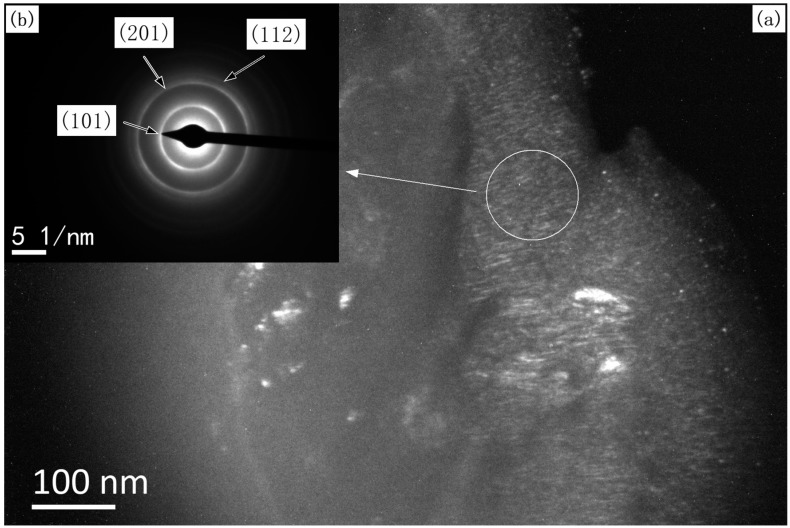
TEM images of the synthesized TiB_2_ film formed by CVD with a TiCl_4_/BCl_3_ gas flow ratio of 0.5: (**a**) dark field image, (**b**) corresponding selected-area electron diffraction pattern.

**Figure 6:**
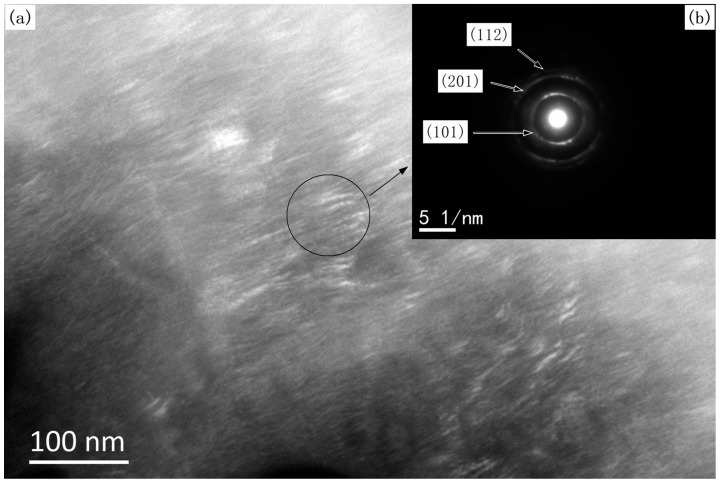
TEM images of the synthesized TiB_2_ film formed by CVD with a TiCl_4_/BCl_3_ gas flow ratio of 1: (**a**) dark field image, (**b**) corresponding selected-area electron diffraction pattern.

The grain size of the TiB_2_ films was calculated by XRD patterns of TiB_2_ films (Figure 2) using the Scherrer formula. The grain size was 58 ± 3 nm when the TiB_2_ film was deposited at a stoichiometric TiCl_4_/BCl_3_ gas ratio of 0.5, and the grain size was 69 ± 3 nm when κ = 1. Hence, all the as-deposited TiB_2_ films had a nano-sized grain structure. Figure 5 and Figure 6 show TEM images of the films formed under different gas flow ratios of TiCl_4_/BCl_3_. Bright nano-sized shapes can be observed in the dark field images (Figure 5a and Figure 6a), wherein it can be seen that the common shapes of the grains reveal a columnar and that the grain size is at the nano scale. The grain size is also easily observed. The grains are larger in Figure 6a than in Figure 5a. Analysis of the selected-area electron diffraction (SAED) pattern (Figure 5b and Figure 6b) revealed only the presence of TiB_2_. The average grain size in the plan view (dark field image) was estimated to be at the nano scale and to cause continuous rings in the diffraction pattern (as shown in Figure 5b and Figure 6b). However, the diffraction ring, as shown in Figure 5b and Figure 6b, is a little bit different. In Figure 5b, the diffraction ring is smooth and continuous. In contrast, the ring intensities in Figure 6b do not show uniform continuity, and there are bright spots on the circular rings. This is because the grain of the selected area in Figure 6a is larger. Thus, the SAED pattern further reveals that the grain size in Figure 6a is greater than that in Figure 5a. These SAED results correspond to Figure 5a and Figure 6a. As a result, TEM investigations indicate that all as-prepared TiB_2_ films are nanocrystalline and that the grain size is different under various TiCl_4_/BCl_3_ gas flow ratios. Obviously, the TEM results are in good agreement with the results calculated by the Scherrer formula. Therefore, TiCl_4_/BCl_3_ gas flow ratio has a certain impact on the grain size of as-prepared TiB_2_ films via CVD. The grain size of TiB_2_ films deposited at κ = 1 is greater than that of other films obtained under conditions where κ = 0.5.

## 4. Conclusions

TiB_2_ was prepared via the vapor-phase reaction of TiCl_4_, BCl_3_, and H_2_ on a high pure graphite substrate. The following information was obtained:

1. TiB_2_ could be deposited at 1000 °C and 10 Pa by a CVD system. All deposits obtained under the condition of excessive hydrogen and different TiCl_4_/BCl_3_ gas flow ratios (1/2 and 1/1) were TiB_2_. Other impurity phases such as TiB were not found.
2. These TiB_2_ films are nanocrystalline with a grain size in the range of 60 nm. All of the TiB_2_ films were typically columnar particles structure, and the columnar particles have a random orientation.
3. X-ray diffraction indicated that all of the as-synthesized TiB_2_ films have a preferential orientation growth in the (100) direction.
4. The TiCl_4_/BCl_3_ gas flow ratio has a certain impact on deposition rate and grain size, but these variations in the gas flow ratio of TiCl_4_/BCl_3_ did not appear to influence the preferred orientation of the deposits. The deposition rate is faster when using a greater TiCl_4_/BCl_3_ gas flow ratio, which in this case was 1/1. Meanwhile, when we provided a stoichiometric TiCl_4_/BCl_3_ gas ratio of 1/2, the grain size of the as-deposited TiB_2_ film was smaller.

## Figures and Tables

**Table 1:** Field of deposition parameters.

Substrate	Graphite
deposition temperature	1000 (°C)
deposition time	3 (h)
vacuum level	10 (Pa)
temperature of H_2_	25 (°C)
pressure of H_2_	0.06 (MPa)
flow rate of H_2_	0.9 (m^3^/h)
temperature of TiCl_4_	135 (°C)
pressure of TiCl_4_	0.1 (MPa)
flow rate of TiCl_4_	0.055, 0.11 (m^3^/h)
temperature of BCl_3_	12 (°C)
pressure of BCl_3_	0.1 (MPa)
flow rate of BCl_3_	0.085 (m^3^/h)

**Table 2:** The EDS (energy dispersive spectroscopy) analysis results of different regions of samples in Figure 3 and Figure 4.

Spot No.	Element	wt %	atom %	Ti/B Atom Ratio
1	B	38.01	73.15	0.367
	Ti	61.92	26.85	
2	B	37.68	72.84	0.373
	Ti	62.32	27.16	
3	B	33.54	69.10	0.447
	Ti	66.46	30.90	
4	B	33.23	68.80	0.453
	Ti	66.70	31.20	
